# Supplementary material for: Developmental and Reproductive Impacts of Four Bisphenols in Daphnia magna
Source: Int J Mol Sci. 2022 Nov 23;23(23):14561. doi: 10.3390/ijms232314561 (PMC9738221; doi:10.3390/ijms232314561)
Supplement: Supplementary file 1 [file ijms-23-14561-s001.zip › ijms-1998237-supplementary.pdf]

Table S1. Abbreviation and description of the parameters in this study

Abbreviation	Description
BPA	bisphenol A
BPS	bisphenol S
BPF	bisphenol F
BPAF	bisphenol AF
CAT	catalase
SOD	superoxide dismutase
MDA	malonaldehyde
cyp314	*cytochrome p450 314*
ecra	*ecdysone receptor a*
ecrb	*ecdysone receptor b*
usp	*ultraspiracle*
ftz-f1	*fushi tarazu factor-1*
cht	*chitinase*
vtg1	*vitellogenin 1*
vtg2	*vitellogenin 2*
vmo1	*vitelline outer layer membrane protein 1*

Table S2. Primers used for qPCR validation

Gene Name	GenBank Accession No.	Primer sequence (5'→3')
β-actin	XM 032925012.2	F: CCCCATTTATGAAGGTTACGC<br>R: CCTTGATGTCACGGACGATT
cyp314	XM 032928208.2	F: ACTATGTATGGACTTCCCTGGTG<br>R: TTATCGCGGGTGTCAACG
ecra	XM 045171284.1	F: CAGTTCGTCCATGTCGATGAG<br>R: CGAAGGCGGTAAGGTAGAATG
ecrb	XM 045171282.1	F: CACCACAACCAACTGCATTTAC<br>R: CCATTAATGTCAAGATCCCACA
usp	XM 032928825.2	F: TAGGCCACTCGGGTTACTTAAA<br>R: GAGTGGGTGGTTAGGTGGATAA
ftz-f1	XM 045177877.1	F: TCTTACCGGACATTCACGCC<br>R: ACAGCCGTTGAGATGCTTGA
cht	XM 032938061.2	F: CAGAGCAAATAGGGCGAGAG<br>R: GCGATTGTGCCGTGTATTT
vtg1	XM 045169126.1	F: GCTACCCACGTCAAGTAATG<br>R: GCTGCCGTAGTCTCAACAGAA
vtg2	XM 045169122.1	F: CACTGCCTTCCCAAGAACAT<br>R: ATCAAGAGGACGGACGAAGA
vmo1	XR 006648693.1	F: TATTACGCGGTTCAGACGTG<br>R: GTTGTCCGCCTCACTACCAT
